# Annotations

**Published:** 1896-01-11

**Authors:** 


### ANNOTATIONS.

### The British Medical Association and Quackery.

THE British Medical Association, in its journal for January 4th, sets forth what it calls a "programme and a policy" for the New Year. Among other things, it takes occasion to praise the action of the General Medical Council in determining in certain cases to undertake prosecutions under the Medical Act, 1858. The Association, it appears, is considering whether or not it can promise a certain amount of co-operation. We cannot help thinking that this is not altogether the position which the British Medical Association should take up at the present period of rapid medical development. The Association is a body composed entirely of volunteers. It has now a comparatively long history behind it, and is still increasing in numbers in almost all its branches, and wherever English men and English doctors are to be found. The reasonable presumption, therefore, is that it commands the full sympathy and confidence, as well as the loyal adhesion, not only of men of all ranks among us, but of the profession itself as a whole. Cannot the Association, then, do something more than merely "report suitable cases" of quackery for prosecution to the General Medical Council? It should be, and must be if it is to survive, the natural defender of the profession against all its enemies. If this view be accepted, the Association should seek to affiliate with itself, and to convert into departments of its own activity, every smaller association which has for its object medical defence, medical protection, or medical inprovement and progress in any form whatever. If this be not done, the smaller associations will tend to do two things: to disintegrate the British Medical Association, and to divide medical men into a number of sects or small bodies, not one of which, by reason of its smallness and impecuniosity, can hope to have any public influence at all commensurate with the real weight of the medical profession as a whole. We commend this view of the case to the leaders of the Association and to the able editor of its widely-read and influential journal.

### Sanitation means Clean Living.

DR. RUSSELL, the senior medical officer of health for Glasgow, has brought together a history of the Evolution of Public Health Administration which is full of interest in many ways. We have not space here to analyse it fully, but we would point out how large a proportion of the most effectual sanitary work that has been done in this century has had to do with the removal of those filth conditions which have led to disease rather than the management of the diseases which have been so produced. The lessening of the death-rate in Glasgow during the last thirty years has been enormous; but this interesting point is to be noted, that much as fevers have diminished it is not in them alone that the saving of life has taken place. Far too much is it the custom to think that sanitarians are concerned chiefly with fevers and such-like disorders. An examination of the tables presented by Dr. Russell shows at once that while under a strict sanitary administration fevers have diminished, the general death-rate also has declined, and that coincidently with the prevention of death from such recognised infectious diseases as fever there has also been a progressive diminution of the mortality from other diseases which are not usually placed in that category, and especially from consumption. Dr. Neil Arnott, describing the condition of Glasgow in 1840, says: "We entered a dirty low passage like a house door, which led from the street through the first house to a square court immediately behind, which court, with the exception of a narrow path around it, leading to another long passage through a second house, was occupied entirely as a dung receptacle of the most disgusting kind. Beyond this court the second passage led to a second square court, occupied in the same way by its dunghill; and from this court there was yet a third passage, leading to a third court and third dunghill. There were no privies or drains, and the dung-heaps received all filth which the swarm of wretched inhabitants could give; and we learned that a considerable part of the rent was paid by the produce of the dung-heaps." If we are to understand at all the immense benefits which have accrued to the country from what is called sanitary progress, it is necessary that we should recognise that the great thing which sanitary authorities have done for us during the last thirty or forty years is not the provision of hospital accommodation, of which just now we hear so much, but the enforcement of cleanliness and decency. These are the true protectives, not merely against a few fevers, but against that wide-spread enfeeblement which enables all sorts of maladies to carry us off before our time.

### Nerves and Noises,

A WEEK or two ago Dr. F. H. Corbyn delivered a lecture, under the auspices of the Association for the Suppression of Street Noises, on the subject of "Noises and Nerves." One would have thought that there could not be two opinions, and as a matter of fact there are not two medical opinions, as to the injuriousness to nerves and health of the babel of confusion which makes some portions of London hideous by day and night. No doubt in a vast population like our own a certain amount of noise and a certain variety of more or less unpleasant sounds are inevitable, and cannot be got rid of. But for this very reason is it all the more imperative that those noises which are not inevitable should and must be got rid of in the sternest and most uncompromising way. In the matter of street noises we affirm that the main facts to be considered are these—that the strenuous toilers with head and hand in London, to the number of tens of thousands, are now daily sacrificed in nerve and health to a few hundreds or thousands at most who mainly prefer loafing to work. This should not be. Physiology demands that it shall not be. An adequate sense of public order declares that it must not be. Just as in old times our forefathers disestablished street beggars, put up street lamps, and made the public thoroughfares passable and safe by day and night, so must we, in our new times, provide for new necessities, and sternly put an end to every unnecessary hindrance to mental quiet, steadiness of nerve, refreshing sleep, and sound working capacity.

